# The effects of propranolol on the biology and Notch signaling pathway of human umbilical vein endothelial cells

**DOI:** 10.1097/MD.0000000000034672

**Published:** 2023-08-11

**Authors:** Shuming Chen, Xuekai Zhao, Junjie Huang, Na Lin, Qianhui Xu, Jianwei Chen, Jianqiang Huang, Lie Wang, Chen Lin, Zaizhong Zhang

**Affiliations:** a Department of General Surgery, 900th Hospital of the Joint Logistics Support Force (Dongfang Hospital of Xiamen University, School of Medicine, Xiamen University; Fuzhou General Hospital of Fujian Medical University), Fuzhou, Fujian, China; b The Second People’s Hospital of Neijiang, Neijiang, Sichuan, China; c Fujian University of Traditional Chinese Medicine, Fuzhou, Fujian, China; d Department of Anesthesia, Fujian Provincial Hospital, Fuzhou, Fujian, China.

**Keywords:** HUVECs, infantile hemangioma, notch pathway, propranolol

## Abstract

Propranolol is the first choice for treating infantile hemangioma (IH). How propranolol works in IH remains unclear. Infantile hemangioma endothelial cells (HemECs) express Notch1, Jagged, Hey1, and other molecules in the Notch pathway, suggesting that Notch pathway-related molecules play an important role in affecting vascular endothelial cell proliferation. Whether propranolol can affect the Notch signaling pathway in IH treatment is unclear.

**Methods:**

We performed this study to observe the effect of propranolol on the expression of Notch signaling pathway molecules in human umbilical vein endothelial cells (HUVECs) and to explore the therapeutic mechanism of propranolol on IH. HUVECs cultured in vitro were exposed to 60, 120, 240, 360, or 480 µM propranolol. The morphological changes of the HUVECs were observed under an inverted microscope. HUVECs proliferation was detected with Cell Counting Kit-8 (CCK-8). The effects of propranolol on HUVECs apoptosis were detected by flow cytometry. The role of Notch in propranolol inhibition of HUVEC proliferation was analyzed with real-time polymerase chain reaction (PCR) and western blotting.

**Results:**

Propranolol reduced HUVECs numbers and altered their morphology. The inhibitory effect of propranolol on cell proliferation was dependent on the reaction time and drug concentration. Propranolol upregulated Jagged1, Notch1, and Hey1 expression and downregulated delta-like ligand4 (DLL4) expression.

**Conclusions:**

Propranolol may play a role in IH treatment by increasing Jagged1 expression in endothelial cells, activating the Notch pathway and inducing the upregulation of the downstream target gene *HEY1*.

## 1. Introduction

Infantile hemangioma (IH) are the most common vascular benign tumor in infants and young children, with an incidence of about 5% and 10%, respectively. Hemangioma growth is characterized by rapid growth in the first year after birth, followed by gradual subsidence. Most children achieve complete regression before the age of 7 years.^[1]^ In the natural course of IH, about 10% of the tumors grow rapidly, which can cause disfigurement, damage functions, and can even be life-threatening.^[2]^ The main cell components of IHs in the increment phase are hemangioma endothelial cells (HemECs) and hemangioma pericytes. HemECs are the main components of neovascularization. Hemangioma pericytes maintain the stability of the lumen structure around the blood vessels, and the tumor body more mature in the regression stage, which is shown by the decrease in cell density and the appearance of orderly lumen structure.^[3]^ The Notch pathway is a conserved ligand–receptor signaling pathway that regulates cell proliferation, differentiation, and maturation, and plays an important role in the development process.^[4]^ In the regression phase of IH, Jagged1 (JAG1) expression is higher than that in the proliferation phase, and Jagged1 can inhibit cell proliferation effectively after activation.^[5,6]^ Propranolol has become the first-line drug for treating IH. However, the specific mechanism of propranolol in IH proliferation and regression has not been fully elucidated. This study was aimed at investigating the effect of propranolol on human umbilical vein endothelial cells (HUVECs) proliferation in vitro and Jagged1 and Hey1 expression in HUVECs before and after propranolol treatment, and to explore the mechanism of propranolol in IH treatment.

## 2. Materials and methods

### 2.1. Cells

**Figure 1.**
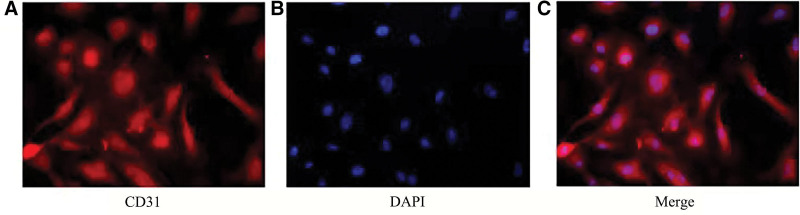
HUVECs immunofluorescence results (×400 magnification). (A) Immunofluorescence showed that the cells were CD31-positive, which conformed with the characteristics of endothelial cells and showed that the cells could be used for subsequent experiments. (B) 4’,6-Diamidino-2-phenylindole staining of HUVECs DNA. (C) Red fluorescence indicates CD31 positivity; the positive rate was > 90%, that is, cell purity was > 90%. HUVECs = human umbilical vein endothelial cells.

Primary HUVECs were purchased from Wuhan Procell Immunofluorescence identification showed that the cells were CD31-positive (Fig. 1), which conformed with the characteristics of endothelial cells and showed that they could be used for subsequent experiments.

### 2.2. HUVEC culture

According to the manufacturer instructions, HUVECs were cultured using vascular cell basal medium and an EC growth kit. Microscope observation showed that the cells grew in a single layer adherently, and the medium was changed every other day. When cell density was 70% to 80%, the cells were passaged at a ratio of 1:2 or 1:3 after trypsin digestion and placed in a CO_2_ constant-temperature incubator. In subsequent experiments, generation 2 to 3 cells were used.

### 2.3. Morphological observation of HUVECs

Second-generation HUVECs were inoculated into 6-well plates after trypsin digestion and cultured with complete medium for 24 hours. Propranolol hydrochloride powder (Sigma, 500 mg) was diluted with complete culture medium to 120, 240, 360, or 480 µM propranolol solutions. When the cell density was about 90%, the cells were incubated with 120, 240, 360, or 480 µM propranolol solution for 12, 24, or 48 hours. The blank control well contained no propranolol; cell morphological changes were observed under an inverted microscope and photographed.

### 2.4. Cell proliferation assessment

HUVECs were seeded in 96-well plates (5 × 10^4^ cells/mL, 100 µL per well, about 5000 cells). The Cell Counting Kit-8 (CCK-8) (Shanghai Biyuntian) assay was performed according to the manufacturer instructions. After the cells had adhered to the growth, 90 µL propranolol solution (0, 60, 120, 240, 360, 480 µM) was added to each well for 12, 24, and 48 hours; 6 wells were used for each group. Then, 10 µL CCK-8 solution was added to each well and incubated in a CO_2_ constant-temperature incubator for 1 hours. The absorbance (optical density [OD]) of each well at a wavelength of 450nm was detected using a microplate reader (American Molecular Devices).

### 2.5. Annexin V–FITC/PI double staining for detecting apoptosis

Logarithmic growth cells were collected, and 1 × 10^6^ cells were inoculated into each well of a 6-well culture plate. Propranolol solution (240 µM) was added to each well and the cells were cultured in a CO_2_ constant-temperature incubator. After 12, 24, and 48 hours, apoptosis was detected with an annexin V–FITC/PI (fluorescein isothiocyanate/propidium iodide) double staining kit (BD Biosciences). The results were analyzed using a BD Biosciences FACSCalibur system.

### 2.6. RNA extraction and quantitative real-time polymerase chain reaction (PCR)

Set group as before. The mRNA of the target genes expressed in the HUVECs was isolated using TRIzol (Invitrogen) and reverse-transcribed into complementary DNA (cDNA), which was stored at −80°C for use. The housekeeping gene *GAPDH* was used as the internal reference gene. The cDNA was detected by fluorescence quantitative PCR (Bio-Rad) according to the kit instructions. The Notch signaling gene sequences were obtained from the National Center for Biotechnology Information Gene Library. The primers are listed in Table 1.

**Table 1:** Primer sequences.

Gene		Primer sequence
Notch1	Forward	GAGGCGTGGCAGACTATGC
	Reverse	CTTGTACTCCGTCAGCGTGA
Jagged1	Forward	GTCCATGCAGAACGTGAACG
	Reverse	GCGGGACTGATACTCCTTGA
DLL4	Forward	GCCCTTCAATTTCACCTGGC
	Reverse	CAATAACCAGTTCTGACCCACAG
Hey1	Forward	GTTCGGCTCTAGGTTCCATGT
	Reverse	CGTCGGCGCTTCTCAATTATTC
GAPDH	Forward	CTGGGCTACACTGAGCACC
	Reverse	AAGTGGTCGTTGAGGGCAATG

DLL4 = delta-like ligand4.

### 2.7. Western blotting

After 12, 24, and 48 hours propranolol intervention, cellular protein was extracted from the cell lysate and stored at −80°C. Bicinchoninic acid working solution was prepared according to the instructions, 2 µL was added to each well, incubated in a constant-temperature box for 30 minutes, and the protein concentration extracted was detected by an enzyme labeling instrument. The protein sample was separated by 10% sodium dodecyl sulfate–polyacrylamide gel electrophoresis, transferred to nitrocellulose membranes, sealed, and rabbit anti-human primary antibody was added. The primary dilution ratio was as follows: Notch1 (1:1000, ABclonal, A19090), JAG1 (1:800, ABclonal, A12733), delta-like ligand4 (DLL4) (1:800, ABclonal, A19090, A12943), HEY1 (1:800, ABclonal, A19090, A16110), and GAPDH (1:2000, TransGen Biotech). After the membranes had been washed, the secondary antibody (1:1000 diluted horseradish peroxidase coupled with goat anti-mouse IgG) was added, and the membranes were incubated on a shaking table at room temperature for 1h. Color development and imaging were performed with a fully automated chemiluminescence image analysis system.

### 2.8. Statistical analyses

All values are expressed as the mean ± standard deviation; the statistical significance between groups was analyzed with one-way analysis of variance, followed by Tukey post-test. The difference was considered statistically significant when *P* < .05.

## 3. Results

### 3.1. Effect of propranolol on HUVECs morphology

**Figure 2.**
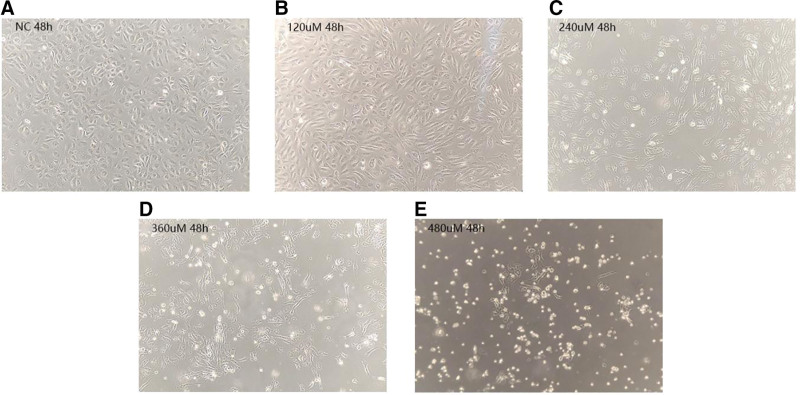
HUVECs morphological changes following 48 h propranolol treatment (×100 magnification). HUVECs = human umbilical vein endothelial cells.

Observed under an inverted microscope, the control cells had regular morphology, consistent size, paving stone-like arrangement, and few cell deaths within 48h. As the propranolol concentration increased and the duration of action was prolonged, cell damage continued to increase: The cells gradually change from diamond-shaped or oval shapes with antennae in the adherent state to round shapes without antennae, the cell volume decreased, and the cell membrane became blurred; the number of dead cells gradually increased, which showed that the number of cell attachments had decreased, and the suspension of high-brightness cells was increased (Fig. 2).

### 3.2. Effect of propranolol on HUVECs proliferation

**Figure 3.**
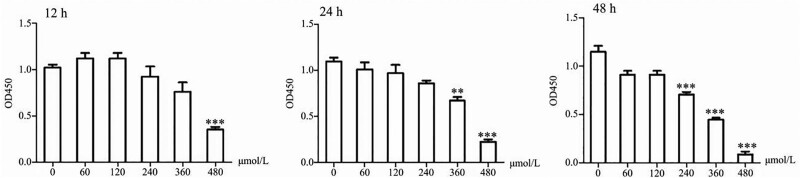
CCK-8 detection of the effect of propranolol on HUVECs proliferation. Data are presented as the mean ± SD (n = 3, *P < .05, **P < .01, ***P < .001). CCK-8 = Cell Counting Kit-8, HUVECs = human umbilical vein endothelial cells.

The cell proliferation in each of the 6 groups at 12, 24, and 48 hours after inoculation was detected with CCK-8 solution. Under the same concentration of propranolol, the inhibition of HUVECs proliferation was gradually enhanced by increasing the drug duration of action. During the same intervention duration, increasing the propranolol concentration led to more obvious downregulation of cell proliferation. There was no significant difference in cell numbers at 60, 120, and 240 µM propranolol within 24 hours (*P* > .05). The cells in the 360 and 480 µM propranolol groups decreased significantly at 48 hours, and the difference was statistically significant (*P* < .05) (Fig. 3). The results show that propranolol can inhibit HUVECs proliferation; increasing the propranolol concentration or the duration of action leads to a more significant inhibitory effect.

### 3.3. Effect of propranolol on HUVECs apoptosis

**Figure 4.**
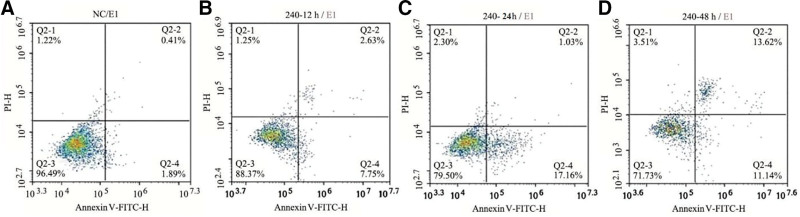
Flow cytometry detection of the effects of propranolol on HUVECs apoptosis. HUVECs = human umbilical vein endothelial cells.

The effect of the propranolol solution on HUVECs apoptosis was measured with flow cytometry, which was performed after FITC–annexin V/PI double staining. After the propranolol exposure, HUVECs apoptosis became more obvious with the prolongation of culture time (*P* < .05) (Fig. 4).

### 3.4. Detection of Notch signaling molecule gene expression

**Figure 5.**
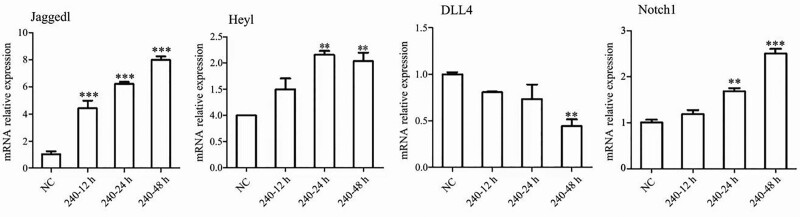
RT-PCR detection of Notch pathway molecule mRNA expression in HUVECs. Data are presented as the mean ± SD (n = 3, *P < .05, **P < .01, ***P < .001). HUVECs = human umbilical vein endothelial cells, RT-PCR = real-time polymerase chain reaction.

The expression of Notch-related genes in HUVECs after 12, 24, and 48 hours intervention with 240 µM propranolol solution was detected with real-time fluorescence quantitative PCR. *JAG1, NOTCH1*, and *HEY1* mRNA expression increased gradually (*P* < .05), and *DLL4* mRNA expression was downregulated (*P* < .05) (Fig. 5).

### 3.5. Detection of Notch signaling molecule protein expression

**Figure 6.**
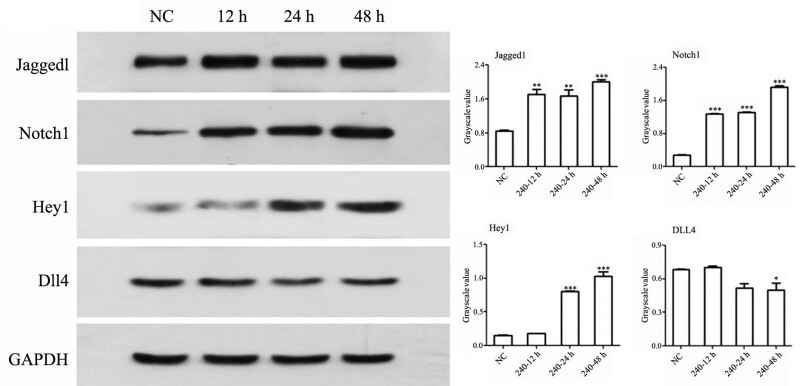
Western blot detection of Notch pathway protein expression in HUVECs. Data are presented as the mean ± SD (n = 3, *P < .05, **P < .01, ***P < .001). HUVECs = human umbilical vein endothelial cells.

The changes in Notch signaling protein expression at 12, 24, and 48 hours were detected with western blotting. JAG1, NOTCH1, and HEY1 protein expression was increased and DLL4 expression was decreased with the prolongation of the duration of action, and the difference was statistically significant (*P* < .05) (Fig. 6).

## 4. Discussion

In this study, we found that the effective concentration of propranolol was 240 µM, Figure 2: the treatment of higher concentrations (360, 480 µM) showed more proliferation compared to the treatment of 240 µM. We found that the endothelial cells continued to grow in the absence of propranolol intervention or under low-concentration propranolol intervention; at 240, 360, and 480 µM propranolol, we obtained a very good inhibitory effect. The results show that the inhibitory effect of the propranolol solution on the vascular endothelial cells was concentration and time-dependent, and that 360 and 480 µM propranolol had a killing effect. After 48 hours, the number of living cells in high-propranolol concentrations were not obviously different between the groups; 240 µM propranolol had a very good time–effect curve. We deemed it unnecessary to observe the cells to the end of the 48 hours, as the low number of living cells and influence on the expression of molecule quantity showed that the action of propranolol on Notch molecular expression change is of great significance. The pathogenesis and regression of IH involves multiple signaling pathways in the human body, and includes the vascular endothelial growth factor (VEGF), Notch, and mTOR signaling pathways, but the molecular mechanisms involved remain unclear. Multiple signaling pathways may be activated or inhibited at high concentration, which accelerates endothelial cell apoptosis. Moreover, the interaction between the multiple pathways of propranolol in the treatment mechanism of IH is also worth studying.

Propranolol is a nonselective β1 and β2 adrenergic receptor blocker that has long been used for treating cardiovascular diseases. In 2008, Léauté-Labrèze^[7]^ inadvertently found that propranolol resolves hemangioma in children. Subsequently, several studies confirmed the efficacy and safety of propranolol for treating IHs; propranolol, instead of glucocorticoids, gradually become the first-line drug for treating hemangiomas. However, the mechanism of propranolol in IH treatment is unclear. At present, it is believed that the mechanisms of propranolol in the regression of IH include vasoconstriction, inhibition of angiogenesis, and promotion of apoptosis. The microcirculation capillaries of normal tissues contain β receptors, and propranolol can constrict blood vessels. Propranolol can downregulate VEGF signal transduction in IH, and the VEGF pathway is one of the important means of regulating vascular development in vivo.^[8]^ Meanwhile, propranolol can downregulate MMP2 and MMP9 expression to reduce the invasiveness of vascular endothelial cells.^[9]^ Propranolol has an anti-angiogenic effect on HUVECs, and at the same time can inhibit vascular endothelial proliferation and differentiation.^[10]^ Here, we found that propranolol inhibited endothelial cell proliferation while promoting apoptosis effectively. Lower propranolol concentrations (60, 120, 240 µM) caused different HUVECs morphological changes, and the cell survival number did not change significantly. By contrast, higher propranolol concentrations (360, 480 µM) caused abrupt change, and the cell survival rate decreased significantly.

On the vascular bed, microvessels form blood vessels in a budding or dividing manner. The signs of proliferation, migration, and remodeling of differentiated and mature vascular endothelial cells are the formation of new blood vessel branches and capillary plexus.^[11]^ The Notch signaling pathway plays an important role in normal embryonic development, angiogenesis, tumorigenesis, and other pathophysiological processes.^[12]^ In mammals, the Notch family consists of 3 receptors (Notch1, Notch3, Notch4) and 4 ligands (DLL1, DLL4, Jagged1, Jagged2). Jagged1, an important ligand in the Notch signaling pathway, is mainly expressed in vascular endothelial cells and can also be expressed in smooth muscle cells, which plays an important role in angiogenesis.^[13]^ Inhibiting Notch signaling can improve endothelial cell proliferation during budding. If Notch signaling is activated, endothelial cell proliferation will be inhibited. In proliferative IH, endothelial cells expressing Jagged1 can induce hemangioma progenitor cells to transform into pericytes and promote vascular maturation. In an IH mouse model, blocking Jagged1 inhibited angiogenesis.^[14]^ *HEY1* is an important downstream target gene of the Notch pathway, and Hey1 is expressed in the endothelial cells of hemangioma.^[15]^ Further, knockout of the effector genes *Hesr1*/*Hey1* and *Hesr2*/*Hey2* of the Notch pathway caused severe vascular defects and bleeding in mouse embryos, and death before maturation.^[16]^ In the present study, propranolol intervention inhibited HUVEC proliferation and increased Jagged1 expression in a time-dependent manner. We had speculated that propranolol may inhibit endothelial cell proliferation by upregulating Jagged1. In our experiments, we found that propranolol inhibited Notch1 expression in the HUVECs, which is consistent with the results of Sun et al.^[9]^ In addition, we found that Hey1 expression in the downstream target of the Notch pathway was upregulated after the propranolol intervention, which suggests that Jagged1 may activate the Notch pathway and induce downstream Hey1 signal activation, thereby inhibiting endothelial cell growth.

By observing the inhibitory effect of propranolol on HUVECs and detecting the intervention-induced changes in Notch signaling molecule expression, we provide a preliminary description of the role of the Notch signaling pathway in propranolol treatment of IH, which provides a new idea for diagnosing and treating IH, evaluating the curative effect, and developing new drugs. However, our study has some shortcomings. First, there was no direct correlation analysis between Jagged1 and Hey1, and the possibility of other signaling pathways cannot be ruled out; therefore, it is necessary to further study the effect of knockout or inhibition of the *JAG1* gene on the downstream target gene *HEY1* and the interaction of the Notch pathway with other signaling pathways to improve the mechanism of propranolol in IH treatment. Second, in this study, we found that the effective concentration of propranolol was 240 µM, which was different from the clinical effective concentration, because this concentration was specific to HUVECs. Third, due to easy access and high purity, HUVECs are a valuable model for the study of physiopathological processes. HUVECs have been used widely as a model system in vitro, due to their ready availability and demonstrated ability to form neovasculature.^[17-20]^ Accordingly, after we had failed to obtain HemECs from hemangioma cases, we decided to use HUVECs to study the cytology of hemangioma. But the absence of HemECs from hemangioma cases is another limitation of our study.

## 5. Conclusion

Propranolol can inhibit HUVECs proliferation and promote HUVECs apoptosis. The Notch signaling pathway, present in HUVECs cultured in vitro, is involved in regulating the biological behavior of endothelial cells. Propranolol upregulated Jagged1, Notch1, and Hey1 expression and downregulated DLL4 expression in the HUVECs. We speculate that propranolol might inhibit the formation of IH by inhibiting endothelial cell proliferation, and the mechanism may be related to the propranolol-induced increase in Jagged1 expression and activation of the Notch1 signal in the endothelial cells, which in turn induces the activation of the downstream target gene *HEY1*.

## Author contributions

**Conceptualization:** Shuming Chen, Jianqiang Huang, Lie Wang.

**Data curation:** Na Lin, Chen Lin.

**Formal analysis:** Chen Lin, Na Lin, Jianwei Chen.

**Funding acquisition:** Shuming Chen, Lie Wang, Zaizhong Zhang.

**Investigation:** Qianhui Xu.

**Methodology:** Junjie Huang, Qianhui Xu, Zaizhong Zhang.

**Project administration:** Junjie Huang, Qianhui Xu, Zaizhong Zhang.

**Resources:** Jianqiang Huang.Supervision: Jianwei Chen, Chen Lin, Zaizhong Zhang.

**Validation:** Shuming Chen, Xuekai Zhao, Junjie Huang, Na Lin.

**Writing – original draft:** Shuming Chen, Xuekai Zhao.

**Writing – review & editing:** Lie Wang, Chen Lin.
